# Study on gas-water two-phase seepage law and flow characteristics of the matrix-fracture system in shale gas reservoirs

**DOI:** 10.1371/journal.pone.0337079

**Published:** 2026-01-20

**Authors:** Sijie He, Feifei Fang, Jin Chang, Xizhe Li, Ruilan Luo, Jie Zhang

**Affiliations:** 1 School of Energy Resources, China University of Geosciences (Beijing), Beijing, China; 2 School of Petroleum Engineering, Chongqing University of Science and Technology, Chongqing, China; 3 Research Institute of Petroleum Exploration and Development, Beijing, China; 4 Institute of Porous Flow and Fluid Mechanics, University of Chinese Academy of Sciences, Langfang, China; China University of Petroleum Beijing, CHINA

## Abstract

As a clean energy source, shale gas plays a vital role in supporting China’s strategic objectives of carbon peaking and carbon neutrality through its efficient development. Due to the low porosity, low permeability, complex pore structure, and strong heterogeneity of deep shale gas reservoirs, the gas-water two-phase seepage law and flow characteristics remain unclear. This study focuses on deep shale gas in the Western Chongqing block as the research subject. Through the gas-water displacement experiments combined with NMR techniques, the gas-water two-phase seepage laws and flow characteristics of the shale gas matrix-fracture system were revealed. The results show that: (1) In the water displacement gas experiment, formation water cannot break through the matrix-type sample, with its displacement velocity decreasing over time and eventually approaching zero. Under the same time conditions, the higher the displacement pressure, the faster the displacement velocity, and the higher the NMR signal. The matrix-fracture type sample breaks through in a short time, and the time when the NMR signal is stable is much less than that of the matrix-type sample. The displacement pressure is negatively correlated with the NMR signal. (2) In the reverse gas displacement experiment, the gas cannot break through the matrix-type sample. For the matrix-fracture type sample, as the displacement pressure increases, the water phase relative permeability increases, the irreducible water saturation decreases, the gas phase relative permeability decreases, the co-permeability zone slightly expands, and the equal permeability point shifts to the lower left. The findings provide a theoretical basis for the efficient development of shale gas in China.

## 1. Introduction

China possesses abundant shale gas resources with significant potential for exploration and development [1]. It represents a critical breakthrough for advancing the unconventional energy revolution and serves as an important strategic direction for ensuring national energy security [2]. Accelerating the exploration and development of shale gas is a strategic measure to implement the guiding spirit, such as “vigorously advancing oil and gas exploration and development” and “the rice bowl of energy must be in our own hands [3].” The shale gas in the Wufeng-Longmaxi formations, with a burial depth of less than 3500 m in the south Sichuan area, has achieved economies of scale development. In particular, the Changning, Weiyuan, and Fuling blocks aim to maintain long-term stable production through three-dimensional development, repeated fracturing, and rolling-edge extension [4]. The geological resources of shale gas in China are 123.01 × 10^12^ m^3^, with the geological resources of deep shale gas totaling 55.45 × 10^12^ m^3^. The Sichuan Basin and its surrounding areas account for more than 60% of this total [5]. The evaluation and increase of production for deep shale gas in the Zigong, Luzhou, Yibin, and Dazu blocks are being actively promoted, positioning them as key strategic areas for China’s increase in shale gas reserves and production [6].

Yang et al. [7] believed that some scholars regarded the core as a “black box” and only explored the influence of macroscopic parameters on the gas-water two-phase flow, while neglecting to characterize the fluid flow characteristics within the pore space. NMR is a non-destructive, fast, non-toxic, non-invasive technique, which has been widely applied in unconventional reservoirs in recent years. This technique enables the study of the fluid distribution and flow characteristics in different pore scales [8]. Xue et al. [9] analyzed the NMR imaging and *T*_2_ spectrum, and divided the pores of coal rock into three categories: adsorption pore, percolation pore, and migration pores. It is pointed out that adsorption pores contribute the most to porosity, but contribute less to the flow of the helium-water system. Lin et al. [10] conducted shale spontaneous imbibition and reverse displacement experiments, utilizing the NMR techniques to dynamically monitor the volume distribution of fracturing fluid in pores with varying pore sizes. Studies have shown that there is a pore size threshold in shale gas reservoirs. When the fracturing fluid enters a pore smaller than this threshold, it is difficult to displace effectively during the flowback process. In addition, the study also found that high-salinity fracturing fluid can reduce the volume of fracturing fluid entering below the threshold, thereby increasing flowback efficiency. Based on fractal theory and NMR techniques, Ge et al. [11]studied the microscale flow characteristics of gas-liquid two-phase systems in coal. The study found that in the gas displacement water process, water preferentially occupies the narrow throat and pore wall, while gas displaces water along the center of the large pores. Residual water is mainly distributed in narrow throats, pore blind ends, and large pores connected by narrow throats. Liu et al. [12] combined gas-water flow experiments with NMR techniques to study the gas-water seepage characteristics of tight sandstone, taking into account the dynamic pore throat structure. The results revealed that as pore pressure decreases during the reservoir development process, the pore throats contract, exhibiting strong stress sensitivity, which results in a reduction in both gas and water flow capacities. Furthermore, the influence of dynamic pore throat structures on gas-water flow characteristics becomes increasingly significant as core permeability increases. Chen et al. [13] designed a set of experimental methods and equipment based on NMR techniques for visual monitoring and quantitative evaluation of reservoir damage caused by working fluids, realized the visual monitoring of the whole process of working fluid invasion damage, and quantitatively evaluated the invasion rate, liquid phase saturation and damage degree. Only from the perspective of pore size, in the early stage of working fluid invasion, the working fluid fills both small pores and large pores. In the later stage of working fluid invasion, the filling rate of working fluid in small pores slows down, mainly filling large pores. In general, experimental studies of gas-water two-phase seepage in unconventional reservoirs primarily focus on tight sandstone reservoirs. In contrast, the experimental study of gas-water two-phase seepage in shale reservoirs is relatively limited. Literature reviews indicate that combining NMR techniques with gas-water two-phase seepage experiment is conducive to revealing the gas-water two-phase seepage law and flow characteristics of shale reservoirs.

Therefore, this study focused on the deep shale gas reservoirs in Western Chongqing, selected different types of shale samples, combined with the NMR techniques, conducted water displacement gas experiments under different displacement pressures, and revealed the gas-water two-phase seepage law of the shale gas matrix-fracture system. Reverse gas displacement experiments under different displacement pressures were conducted to clarify the gas-water two-phase flow characteristics of the shale gas matrix-fracture system. The research results are of great significance to the efficient development of shale gas in China.

## 2. Experimental Materials and methods

### 2.1. Experimental materials

**Fig 1:**
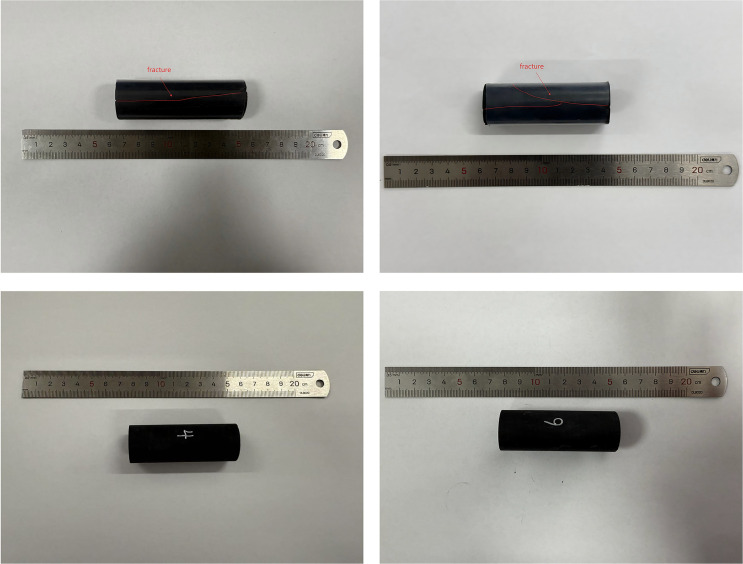
Experimental core photos.

The experimental samples were collected from the Western Chongqing block, a total of four shales. Samples 4# and 6# are matrix-type, while Samples 1# and 3# are matrix-fracture type. The porosity of the matrix-type samples is 1.340% and 1.470%, with an average of 1.405%, while the permeability is 0.0067 × 10^−3^ μm^2^ and 0.0072 × 10^−3^ μm^2^, with an average of 0.0070 × 10^−3^ μm^2^. The matrix-fracture type samples were prepared by artificially fracturing the matrix-type samples. After fracturing, the porosity is 2.252% and 2.266%, with an average of 2.259%, while the permeability is 0.296 × 10^−3^ μm^2^ and 0.276 × 10^−3^ μm^2^, with an average of 0.286 × 10^−3^ μm^2^. The basic parameters of the experimental samples are listed in Table 1, and the experimental sample photos are shown in Fig 1.

**Table 1:** Basic parameters of experimental samples.

Type	Number	Length/cm	Diameter/cm	Permeability/ ×10^−3^μm^2^	Porosity/%
Matrix	4#	7.02	2.52	0.0067	1.340
	6#	7.04	2.52	0.0072	1.470
Matrix-fracture	1#	7.99	2.52	0.296	2.252
	3#	7.48	2.52	0.276	2.266

The experimental water refers to the Chinese standard GB/T 28912−2012 “Determination Method of Relative Permeability of Two-Phase Fluids in Rocks,” using a laboratory-formulated standard formation water with a composition of NaCl: CaCl₂: MgCl₂·6H₂O = 7: 0.6: 0.4 and a density of 1.08 g/cm^3^. High-purity nitrogen gas (99.99%) was used as the experimental gas.

### 2.2. Experimental instrument and scheme

**Fig 2:**
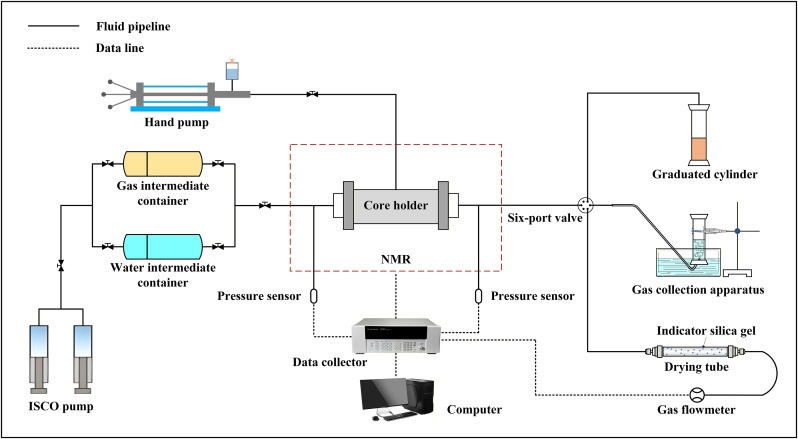
The gas-water displacement experiment flowchart.

**Fig 3:**
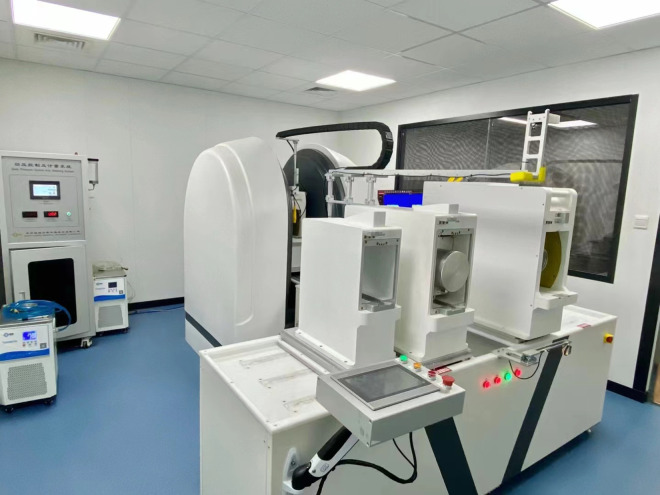
NMR analysis system.

The gas-water displacement experimental instrument for shale samples is shown in Fig 2, and the detailed experimental scheme is presented in Table 2. NMR testing was conducted using a MacroMR12-150H-I large-bore, high-temperature, and high-pressure online NMR imaging analyzer manufactured by Suzhou Niumag Analytical Instrument Corporation (Fig 3). The dominant frequency of the instrument is 12 MHz, employs a rare-earth permanent magnet, and provides a magnetic field strength of 0.3 T ± 0.03 T.

**Table 2:** Experimental scheme.

Experiment	Type	Number	Displacement pressure/MPa	Confining pressure/MPa	Experimental temperature/°C
Water displace gas	Matrix	4#	15	25	Room temperature
		6#	30	40	
	Matrix-fracture	1#	30	40	
		3#	15	25	
Gas displace water	Matrix	4#	25	35	Room temperature
		6#	30	35	
	Matrix-fracture	1#	5	10	
		3#	3	10	

Considering the influence of displacement pressure and sample type, the water displacement gas experiment was conducted. Combined with the NMR techniques, the gas-water two-phase seepage law of the samples under different conditions was analyzed. The water displacement gas experimental system mainly includes the water simulation system, the model system, the NMR analysis system, and the data acquisition system. The water simulation system comprises an ISCO pump and a water intermediate container. The model system includes a core holder, a hand pump, and a graduated cylinder. The NMR analysis system can realize the *T*₂ spectrum and the sectional *T*₂ spectrum test. The data acquisition system consists of high-precision pressure sensors, a data collector, and a computer.

The reverse gas displacement experiment is conducted on samples to study gas-water two-phase flow characteristics. The experimental system for reverse gas displacement is essentially identical to that used for water displacement. The main difference lies in the replacement of the graduated cylinder with a drainage-based gas collection apparatus for matrix-type samples. In contrast, for matrix-fracture type samples, the graduated cylinder is replaced with a drying tube and a gas flowmeter.

### 2.3. Experimental steps

The steps for the water displace gas experiment are as follows:

(1) The sample was placed into the core holder, and confining pressure was applied to the set value using a hand pump.
(2) After the confining pressure stabilized, standard formation water was loaded into the intermediate container and pressurized to the set value using the ISCO pump.
(3) The water simulation system was then connected to the model system. *T*₂ spectra and sectional *T*₂ spectra were measured at regular 24-hour intervals. The tests were conducted in strict accordance with the Chinese standard SY/T 6490−2023 “Laboratory Measurement Specification for Nuclear Magnetic Resonance Parameters of Rock Samples”.
(4) Take out the sample and weigh it.
(5) Repeat the above steps until the displacement velocity is stable, at which point the experiment is considered complete.

The steps of the reverse gas displacement experiment for matrix-type samples are as follows:

(1) The sample was placed into the core holder, and confining pressure was applied to the set value using a hand pump.
(2) After the confining pressure stabilized, high-purity nitrogen was loaded into the intermediate container and pressurized to the set value using the ISCO pump.
(3) The gas simulation system was then connected to the model system. The data were measured at regular 24-hour intervals, including gas production and displacement pressure difference.
(4) Take out the sample and weigh it (water production).
(5) Repeat the above steps until the displacement velocity is stable, at which point the experiment is considered complete.

The steps of the reverse gas displacement experiment for matrix-fracture type samples are as follows:

(1) The sample was placed into the core holder, and confining pressure was applied to the set value using a hand pump.
(2) After the confining pressure stabilized, high-purity nitrogen was loaded into the intermediate container and pressurized to the set value using the ISCO pump.
(3) The gas simulation system was then connected to the model system. The data were measured at regular 10-minute intervals, including gas production, water production, and displacement pressure difference.
(4) Repeat the above steps until the displacement velocity is stable, at which point the experiment is considered complete.

### 2.4. Calculation methods

To quantitatively analyze the displacement velocity of samples during the water displacement gas process, the weighing method was applied. The difference in sample weight between two consecutive time points was measured and divided by the standard formation water density to obtain the invasive water volume. This volume was then divided by the product of the cross-sectional area of the sample and the time interval between the two measurements to calculate the displacement velocity (Equation 1).


$$v=\frac{\left(m_{n}-m_{n-1}\right)}{\rho A\Delta t}$$ (1)

In which: *v*-displacement velocity, cm/d; *m*_*n*_-the weight of the sample at a specific time, g; *m*_*n*-1_-the weight of the sample at the previous time point, g; *ρ-*standard formation water density, taken as 1.08 g/cm^3^; *A*-rock sample cross-sectional area, cm^2^; Δt -interval between consecutive measurements, d.

The weighing method, as described by Equation (2), was applied to calculate the water saturation of different types of samples under varying displacement pressures, thereby studying the variation law of water saturation under different conditions.


$$S_{i}=\left(\frac{m_{w}/\rho }{V_{b}\phi }\right)\times 100\%$$ (2)

In which: *S*_*i*_-saturation, %; *m*_w_-weight of the invaded formation water, g; *ρ*-standard formation water density, taken as 1.08g/cm^3^; *V*_b_-Total volume of shale, cm^3^; *ϕ*-shale porosity, %.

Rui et al. [14] experimentally derived an approximate quantitative relationship between *T*₂ values and pore size in core samples from the Wufeng-Longmaxi Formation (Upper Ordovician to Lower Silurian) in the Zhaotong shale gas demonstration area of the Sichuan Basin. Based on this, and using Equation (3), the classification standard for nanopores established by the International Union of Pure and Applied Chemistry (IUPAC) was converted into T₂-based categories: *T*₂ < 0.1 ms corresponds to micropores, *T*₂ between 0.1 ~ 12.85 ms to mesopores, and *T*₂ > 12.85 ms to macropores.


$$r_{p}=9.1855T_{2}^{0.6637}$$ (3)

In which: *T*_2_-NMR transverse relaxation time, ms; *R*-pore diameter, nm.

Methods for determining gas-water relative permeability curves generally include direct measurement and indirect calculation. While the former can be divided into steady-state and unsteady-state methods, the latter includes the calculation method based on fractal theory, the calculation method based on electrical parameters, and the calculation method combined with the NMR technique [15]. However, most of the indirect calculation methods remain in the laboratory stage. The steady-state method has the drawbacks of a long test cycle, complex experimental operation process, and harsh experimental conditions [16]. The unsteady-state method has been more widely applied in determining gas-water relative permeability curves due to the short test period and simple experimental operation [17]. Comprehensive consideration, the unsteady-state JBN method is used to determine the gas-water relative permeability curve in shale. The equation is as follows [18]:


$$K_{\text{rw}}=f_{\text{w}}\left(S_{\text{g}}\right)\frac{\text{d}[\text{1}/V(t)]}{\text{d}\{\text{1}/[IV(t)}\}$$ (4)

$$K_{\text{rg}}=K_{\text{rw}}\frac{\mu_{\text{g}}}{\mu_{\text{w}}}\frac{\text{1}-f_{\text{w}}\left(S_{\text{g}}\right)}{f_{\text{w}}\left(S_{\text{g}}\right)}$$ (5)

$$f_{\text{w}}\left(S_{\text{g}}\right)=\frac{\text{d}V_{\text{w}}(t)}{\text{d}V(t)}$$ (6)

$$I=\frac{Q(t)}{Q_{\text{0}}}\frac{\Delta p_{\text{0}}}{\Delta p(t)}$$ (7)

$$S_{\text{ge}}=S_{\text{gi}}+V_{\text{w}}(t)-V(t)f_{\text{w}}\left(S_{\text{g}}\right)$$ (8)

In which: *K*_rw_(*K*_rg_)-Water (gas) phase relative permeability, f; *f*_w_(*S*_g_)-Water phase fractional flow, f; *V*(t)-Cumulative gas and water production, dimensionless; *I*-mobility ratio, dimensionless; *μ*_g_-Gas phase viscosity, mPa·s; *μ*_w_-Water phase viscosity, mPa·s; *V*_w_(t)-Cumulative water production, dimensionless; *Q*(*t*)-Core outlet endpoint gas and water rate at time *t*, cm^3^/s; *Q*_0_-Core outlet endpoint water rate at the initial time, cm^3^/s; Δ*P*_0_-Initial pressure difference, MPa; Δ*P*(*t*)-Pressure difference at time *t*, MPa; *S*_ge_-Endpoint gas saturation at the outlet, f; *S*_gi_-Initial gas saturation, f; *S*_g_-Gas saturation, f.

## 3. Results

### 3.1. Study on gas-water two-phase seepage law

#### 3.1.1. Study on the variation law of displacement velocity.

**Fig 4:**
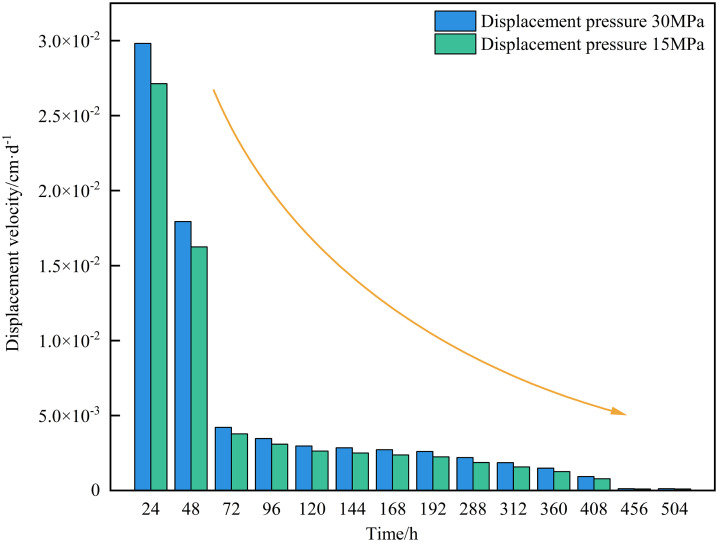
Displacement velocity characteristics of matrix-type samples under different displacement pressure.

As shown in Fig 4, the displacement velocity of the matrix-type sample decreases over time under displacement pressures of 15 MPa and 30 MPa. The higher the displacement pressure, the faster the displacement velocity. In the initial stage of the experiment, invasive water volume was relatively small. Meanwhile, due to the low viscous resistance, water rapidly invaded the core under the combined influence of capillary force and displacement pressure, resulting in a high initial displacement velocity [19]. During the 24 ~ 72 h interval, the increasing viscous resistance caused a marked decline in the displacement velocity. During the 72 ~ 408 h interval, the displacement velocity decreases slowly. During the 408 ~ 504 h interval, the displacement velocity approached zero, signifying the completion of the water displacement gas process. Limited by the experimental sample size, the matrix-fracture type sample experienced breakthrough within a short period, rendering it impossible to calculate its invasion depth and displacement velocity. Based on previous studies on tight sandstones, it can be inferred that under the same displacement pressure conditions, the displacement velocity of the matrix-fracture type sample (high permeability) is faster than that of the matrix-type sample (low permeability) [13].

**Fig 5:**
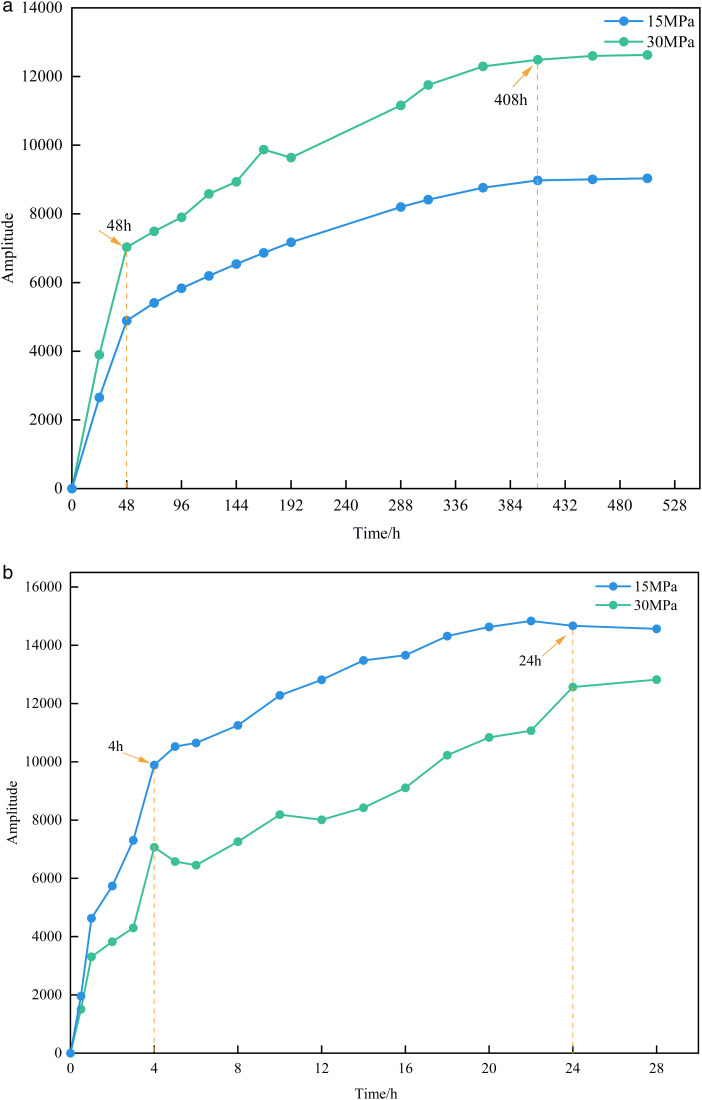
NMR signal variations in different sample types under different displacement pressures.

As shown in Fig 5(a), after subtracting the baseline signal, the NMR signal intensity of the matrix-type sample exhibited an increasing trend over time. During the 0 ~ 48 h interval, the NMR signal increased significantly. During the 48 ~ 408 h interval, the velocity of increase slowed down. During the 408 ~ 504 h interval, the signal intensity approached a stable value and took a relatively long time. The extremely low permeability of this type of sample and higher experimental displacement pressure facilitates the invasion of formation water into the core, and the water cannot break through the core, resulting in a higher NMR signal intensity within the sample. As shown in Fig 5(b), the NMR signal intensity of the matrix-fracture type sample also exhibited an increasing trend over time. Because of the presence of fractures that provided a seepage channel for the water, the water rapidly advanced along the high-permeability fractures, resulting in a breakthrough within a short period. Therefore, the NMR signal intensity of the matrix-fracture type sample is stable during the 24 ~ 28 h interval, which is significantly shorter than that of the matrix-type sample. The matrix-fracture type sample exhibited an opposite trend compared to the matrix-type sample, with lower NMR signal intensity observed at higher displacement pressures. This is attributed to the higher permeability of the matrix-fracture type sample, which allows water to flow rapidly through the core along the fractures. As a result, less water is retained within the sample, leading to a reduction in NMR signal intensity.

**Fig 6:**
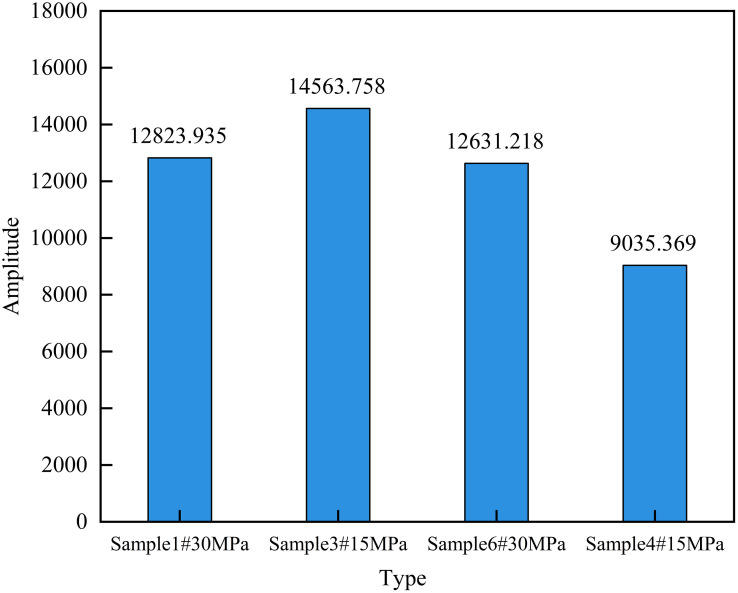
NMR signals of different sample types at the end of the experiment under different displacement pressures.

As shown in Fig 6, when the NMR signal intensity stabilized, the matrix-fracture type sample exhibited consistently higher NMR signal intensity than the matrix-type sample under the same displacement pressure conditions, while requiring less time, reflecting that the displacement velocity of the matrix-fracture type sample is much faster than that of the matrix-type sample.

**Fig 7:**
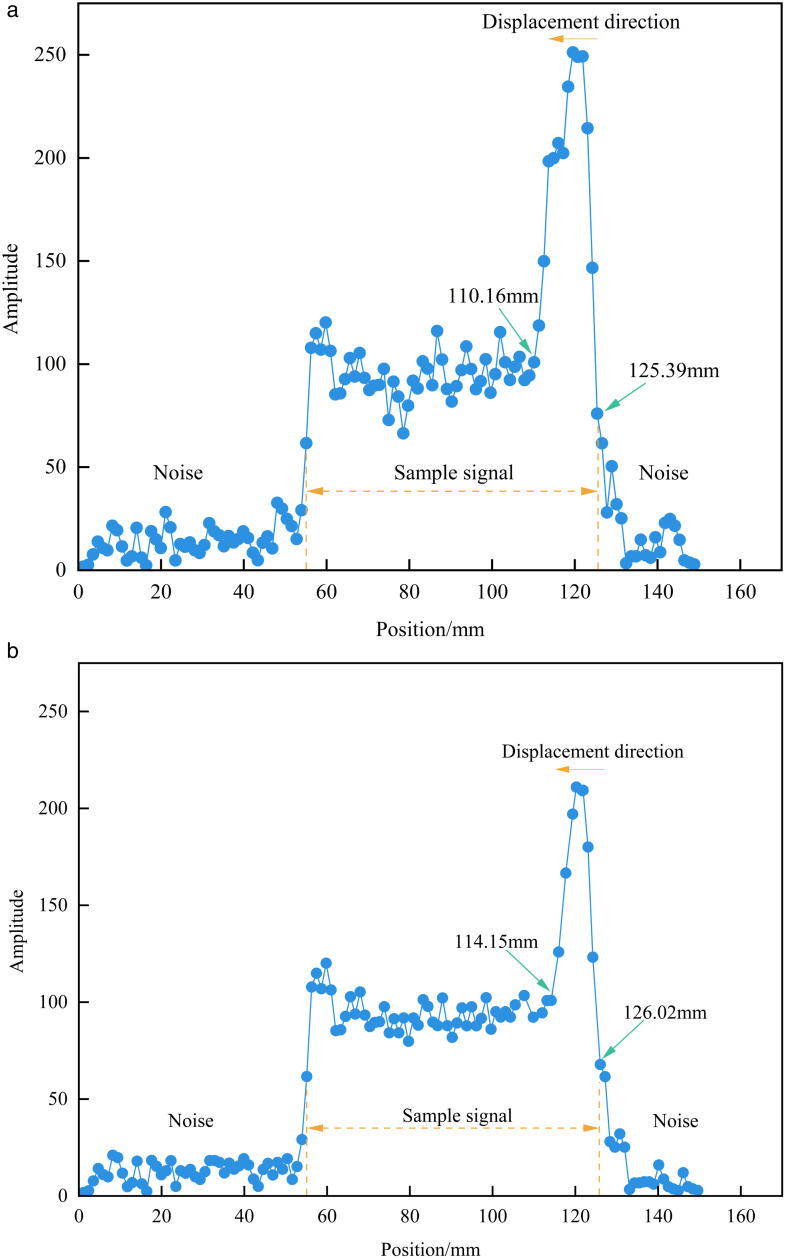
1D frequency encoding.

The SR-CPMG sequence was used to characterize the spatial distribution of signal intensity within the samples and to determine the final invasion depth. At the end of the experiment, the invasion depth of sample 6# was approximately 1.523 cm, accounting for nearly one-quarter of the total sample length (Fig 7a). In comparison, that of sample 4# was approximately 1.187 cm, close to one-fifth of the sample length (Fig 7b). These results indicate that the higher the experimental displacement pressure, the deeper the formation water invades the matrix-type sample.

#### 3.1.2. Study on the variation law of water saturation.

**Fig 8:**
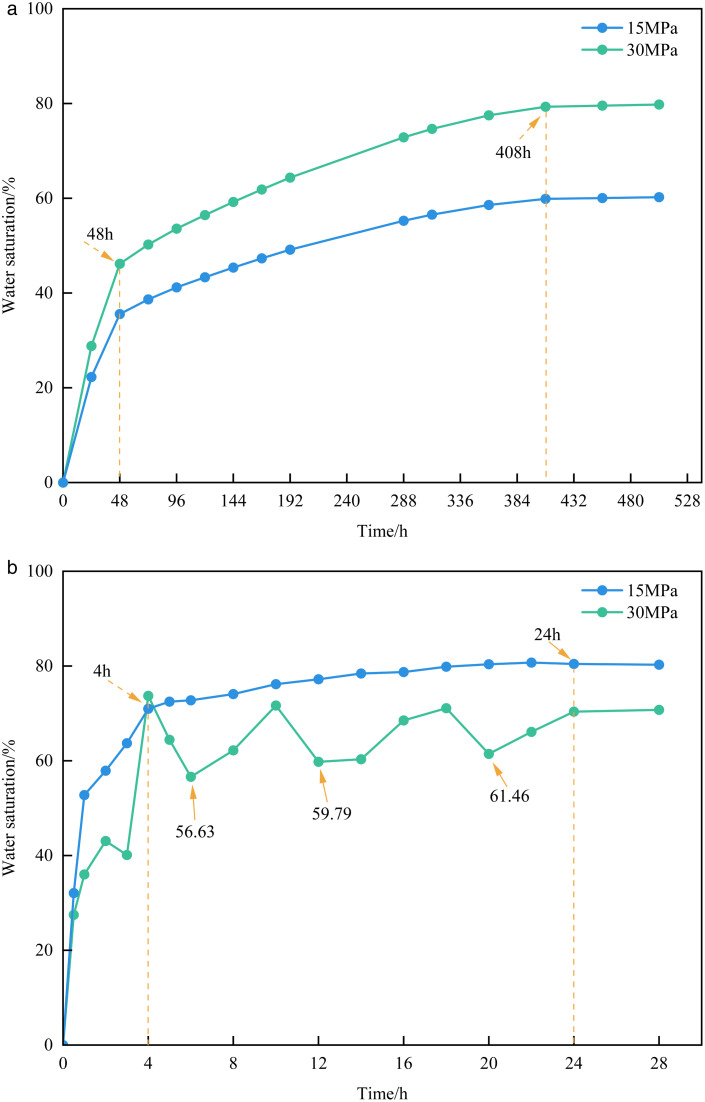
Variation of water saturation in samples under different displacement pressures.

As shown in Fig 8(a), the water saturation of the matrix-type sample increases with time, and higher displacement pressure results in higher water saturation. Consistent with the trend observed in the NMR signal variation, the water saturation increases sharply during the 0 ~ 48 h interval, followed by a slower increase at the 48 ~ 408 h interval, and remains nearly unchanged during the 408 ~ 504 h interval. As shown in Fig 8(b), the matrix-fracture type sample also exhibits an overall increasing trend in water saturation over time; however, under the same conditions, higher displacement pressure corresponds to lower water saturation. Under a displacement pressure of 30 MPa, the water saturation of the matrix-fracture sample exhibits fluctuations. This phenomenon can be attributed to the rapid advancement of the water along the fractures under high pressure, resulting in a significant amount of retained water within the fracture channels in the initial stages. Meanwhile, due to wettability and capillary forces, the water is continuously imbibed from the fractures into the matrix pores and micro-fractures, leading to a rapid increase in water saturation during the initial phase of the experiment. Subsequently, as the displacement time increases, the volume of retained water within the fractures varies, resulting in fluctuating water saturation. A comparison of the saturation drops points of 6 h, 12 h, and 20 h shows that water saturation gradually increases over time, reaching 56.63%, 59.79%, and 61.46%, respectively. This trend further confirms the dynamic process of continuous water imbibition from fractures into the matrix. Under the 15 MPa displacement pressure, the initial stage variation in water saturation mirrors that under 30 MPa, and the water saturation increases sharply. However, due to the slower advancement of water along the fractures under the low displacement pressure, the variation in the retained water volume in the fracture channels is less pronounced. Under the influence of imbibition, water saturation increases more gradually and steadily, with only a slight decline observed between 22 h and 28 h.

#### 3.1.3. Study on the distribution characteristics of formation water.

**Fig 9:**
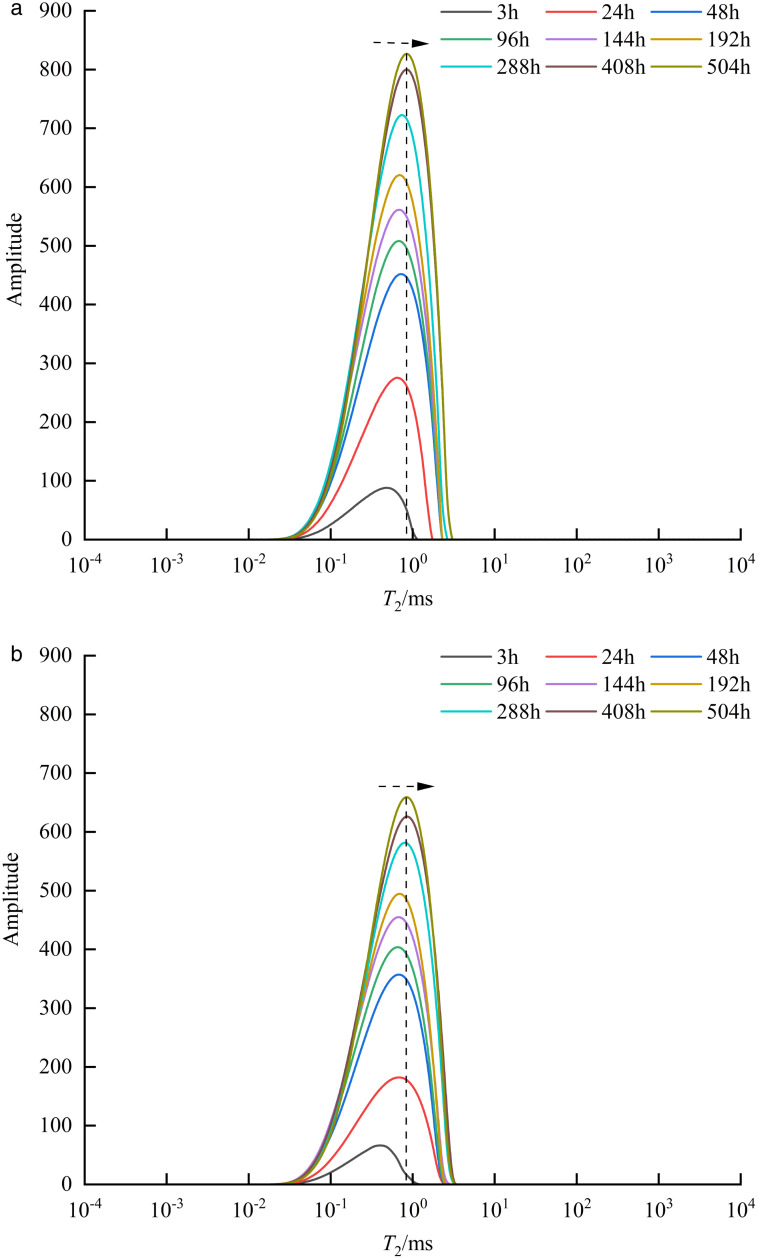
*T*_2_ spectra of matrix-type samples under different displacement pressure.

As shown in Fig 9(a), the *T*₂ spectrum (with baseline signal subtraction) of matrix-type shale sample 6# under a displacement pressure of 30 MPa exhibits a unimodal distribution with good geometric symmetry centered around the peak value, indicating a continuous pore size distribution within the sample [20]. Based on previous classification methods for different pore size ranges, it was found that at 504 h, the *T*₂ values of sample 6# ranged from 0.01 to 3 ms, with *T*_2peak_ intensity of 826.429 and a corresponding relaxation time of 0.87 ms, indicating that the sample is predominantly composed of micropores and mesopores, with minimal development of macropores. With displacement time increased, the overall amplitude of the unimodal *T*₂ spectrum gradually rose, and the *T*₂ corresponding to the peak shifted rightward, indicating that the formation water first filled the micropores and subsequently the mesopores, which can be attributed to the higher capillary pressure in micropore throats compared to mesopores [21]. Moreover, according to the existing research results, it is inferred that water in micropores and mesopores exists in the form of “thick water films” or “water columns” adhering to pore surfaces [22]. As shown in Fig 9(b), the *T*₂ spectrum (with baseline signal subtraction) of matrix-type shale Sample 4# under a displacement pressure of 15 MPa also displays a unimodal distribution. With increasing displacement time, the variation trend of the *T*₂ spectrum is generally consistent with that observed under 30 MPa. However, the *T*₂ peak values at corresponding time points are lower than those at 30 MPa, indicating that for matrix-type samples, higher displacement pressure results in higher *T*₂ peak values.

**Fig 10:**
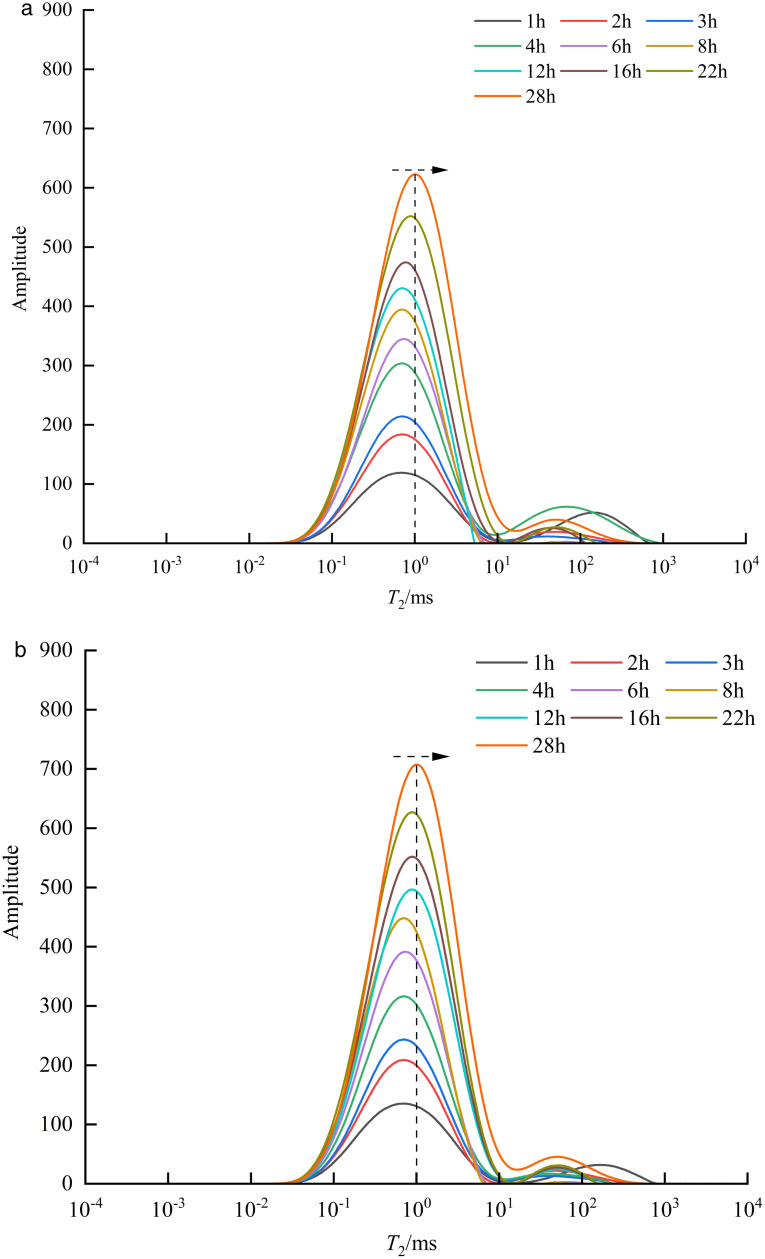
*T*_2_ spectra of matrix-fracture type samples under different displacement pressure.

As shown in Fig 10(a), the *T*₂ spectrum (with baseline signal subtraction) of matrix-fracture type shale sample 1# under a displacement pressure of 30 MPa exhibits a bimodal distribution, characterized by a higher left peak than right, indicating a fine-skewed pore size distribution dominated by micropores and mesopores, with a relatively low proportion of macropores [23]. At 28 h, the left peak of the *T*₂ spectrum for sample 1# ranged from 0.01 to 10 ms, with a *T*_2peak_ intensity of 622.623 and a corresponding relaxation time of 1 ms, indicating well-developed micropores and mesopores. The right peak ranged from 10 to 534 ms, with a *T*_2peak_ intensity of 39.831 and a corresponding relaxation time of 50 ms, indicating relatively fewer macropores and microfractures. Studies have shown that two mechanisms jointly control water imbibition in matrix-fracture type shale [24]. On one hand, formation water retained in fractures exists as movable water; on the other hand, water imbibed by the shale matrix exists within the pore spaces as capillary-irreducible water and surface-irreducible water. As displacement time increases, the amplitude of the left *T*₂ peak gradually increases, and the corresponding *T*₂ value shifts rightward, indicating that formation water preferentially fills micropores and then mesopores, occupying pore surfaces in the form of “thick water films” or “water columns.” The amplitude of the right peak fluctuates over time, which is attributed to the dynamic changes in movable water within the fractures. As shown in Fig 10(b), the *T*₂ spectrum (with baseline signal subtraction) of matrix-fracture type shale sample 3# under a displacement pressure of 15 MPa also displays a bimodal distribution. With increasing displacement time, the change trend of the *T*₂ spectrum is generally consistent with that observed under 30 MPa. However, at the same time points, the peak *T*₂ values under 15 MPa are higher than those under 30 MPa. This indicates that, for matrix-fracture type shale samples, lower displacement pressure leads to a higher left peak *T*₂ value.

**Fig 11:**
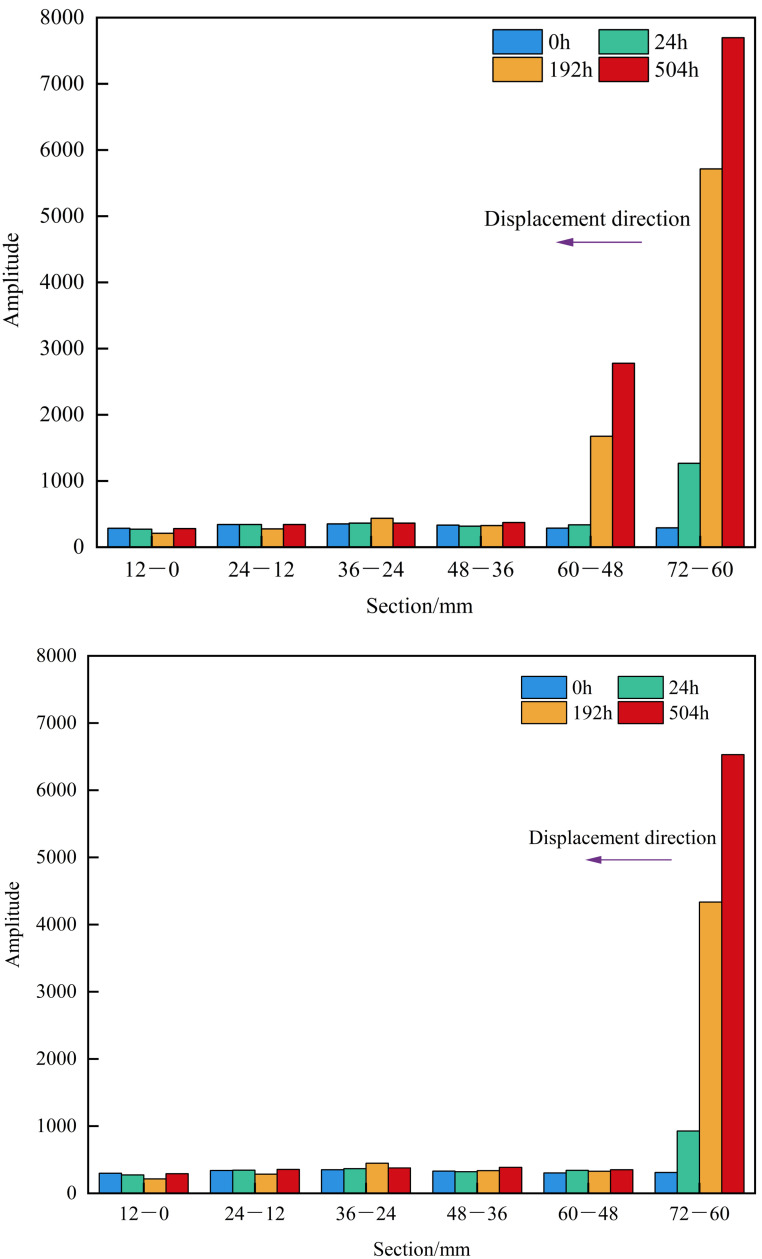
Segmented NMR signal of matrix type samples under different displacement pressure.

**Fig 12:**
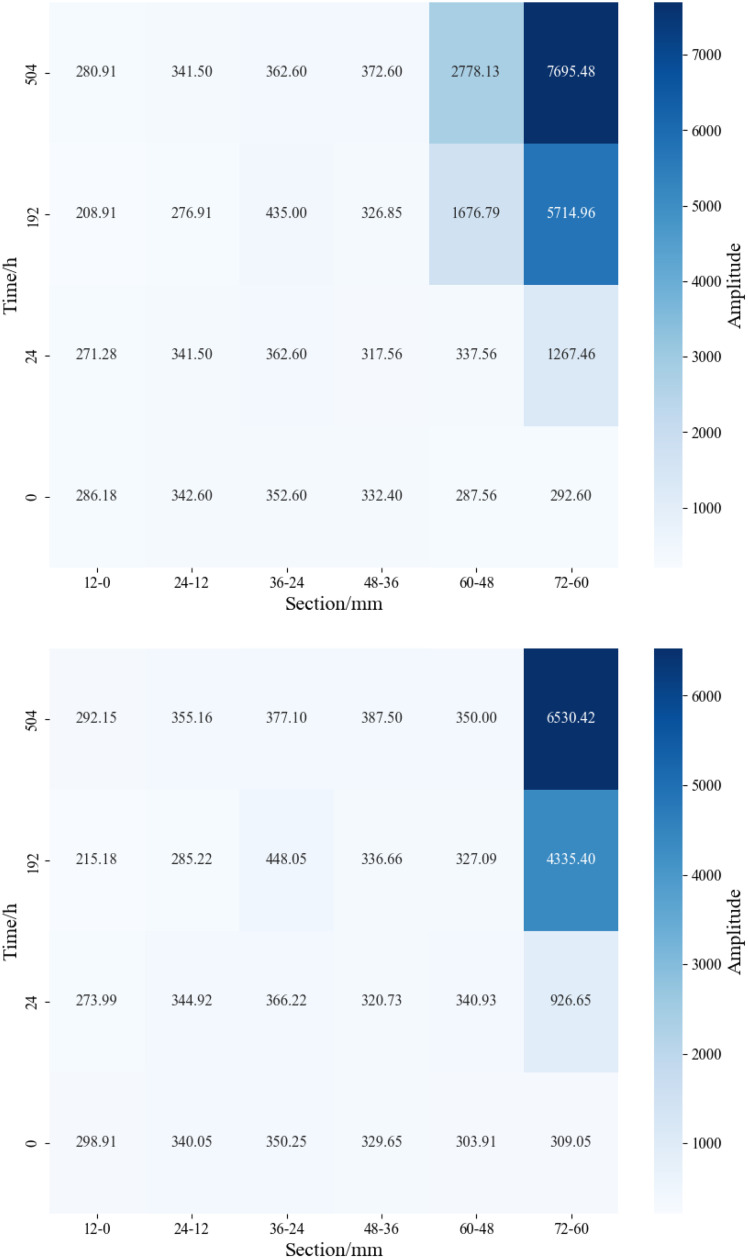
Heatmap of segmented NMR signal for matrix type samples under different displacement pressure.

The lengths of the matrix-type shale samples 4# and 6# are 7.02 cm and 7.04 cm, respectively. For segmented NMR signal detection, the samples were divided into 12 mm × 6 segments. As shown in Figs 11 and 12, detectable NMR signals were observed only at the inlet end of the matrix-type shale, with the signal intensity gradually increasing over time. A higher displacement pressure led to a stronger signal at the same segment for a given time, indicating a greater depth of water invasion. Under the 30 MPa condition, formation water was mainly distributed in the 72−60 mm and 60−48 mm segments; under the 15 MPa condition, it was primarily concentrated in the 72−60 mm segment. This observation is consistent with the findings illustrated in Fig 8, confirming that the water invasion depth remains confined to the frontal end of the matrix-type samples.

**Fig 13:**
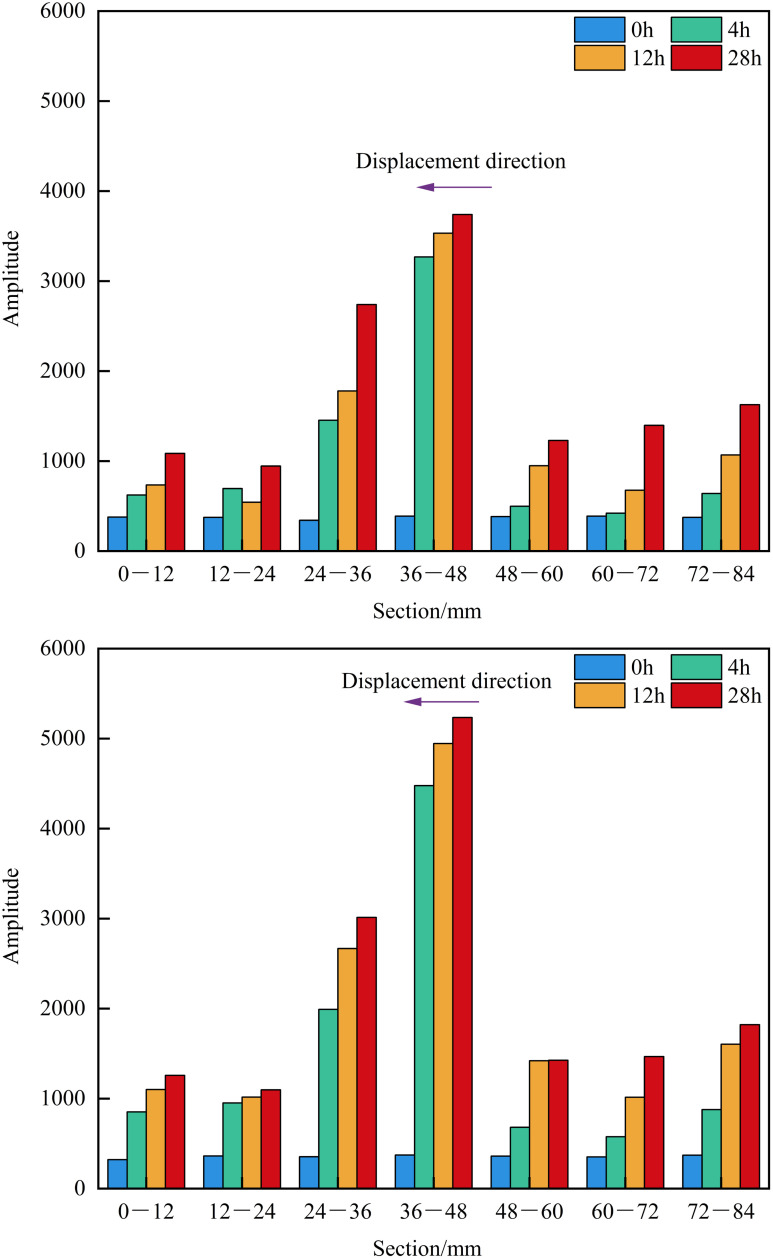
Segmented NMR signal of matrix-fracture type samples under different displacement pressure.

**Fig 14:**
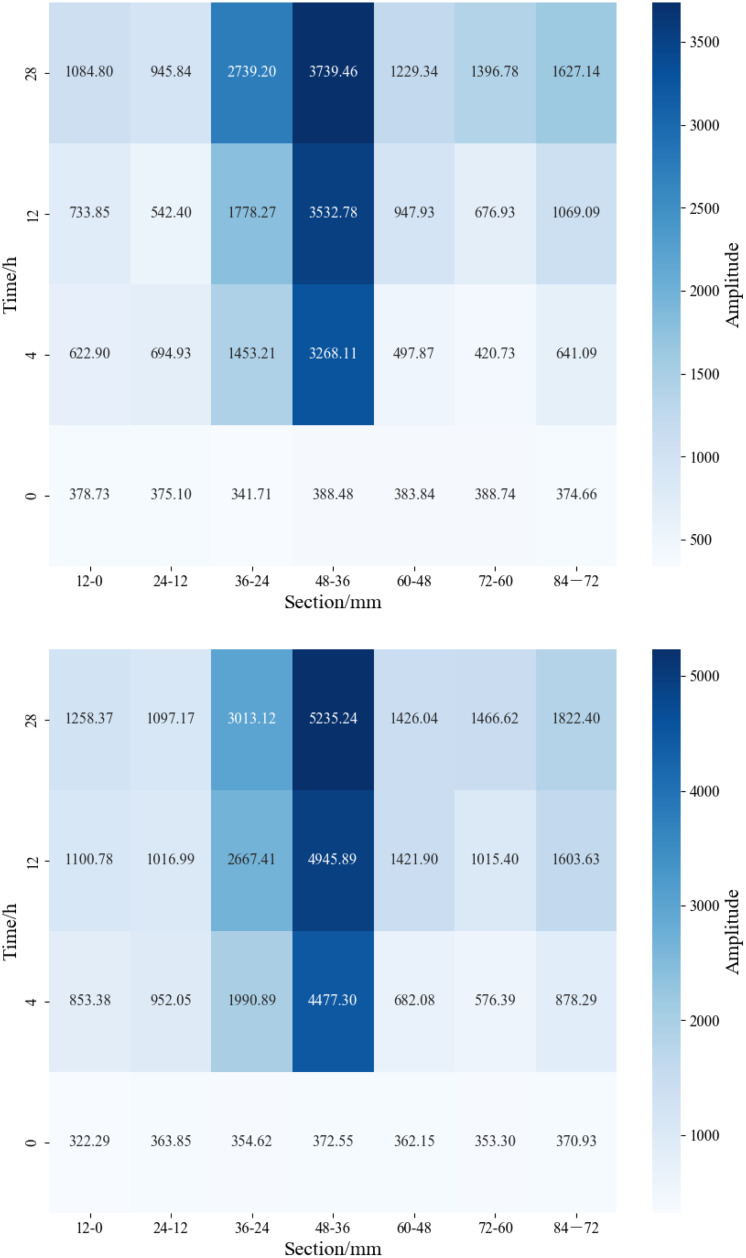
Heatmap of segmented NMR signal for matrix-fracture type samples under different displacement pressure.

The lengths of the matrix-fracture type shale samples 1# and 3# are 7.99 cm and 7.48 cm, respectively. For segmented NMR signal detection, the samples were divided into 12 mm × 7 segments. As shown in Figs 13 and 14, the NMR signal intensity of each segment increased with time. Notably, under lower displacement pressure, the same segment exhibited higher signal intensity at the same time point. After 4 hours of water displacement under 30 MPa, the signal intensity at both ends of the sample became nearly identical, indicating that water had effectively broken through the sample along the fracture. The observation of formation water outflow at the outlet during the experiment also supported this inference. In particular, the segments from 48−36 mm and 36−24 mm showed a sharp increase in NMR signal, indicating that both movable water within the fractures and imbibed water mainly existed in these two sections. As the displacement time increased, the formation water continues to imbibe along the fracture. Except for a slight decrease in signal in the 24−12 mm segment at 12 hours compared to 4 hours, all other segments exhibited a general upward trend in signal intensity. Additionally, the signal at the inlet end was consistently greater than at the outlet end. In the initial stage of displacement (4 h), the signal is predominantly contributed by fluid flow within the fracture network, which can be defined as the fracture-dominated flow stage. In the middle stage of displacement (12 h), the contribution of capillary imbibition of matrix pores to the NMR signal gradually appeared, which can be defined as the start-up stage of matrix pore imbibition. In the late stage of displacement (~28 h), the total water content within the matrix-fracture system reaches a state of dynamic equilibrium, and the NMR signal stabilizes. At the end of the experiment, the highest signal was recorded in the 48−36 mm segment (3739.46), followed by the 36−24 mm segment (2739.20). The signal intensity in the remaining segments, namely 84−72 mm, 72−60 mm, 60−48 mm, 24−12 mm, and 12−0 mm, show basically no difference, further confirming that movable water within the fractures and imbibed water predominantly existed in the 48−36 mm and 36−24 mm segments. Under a displacement pressure of 15 MPa, sample 3# exhibited a similar trend in segmented NMR signal change as sample 1#, with movable water within the fractures and imbibed water mainly existing in the 48−36 mm and 36−24 mm segments.

### 3.2. Study on gas-water two-phase flow characteristics

**Fig 15:**
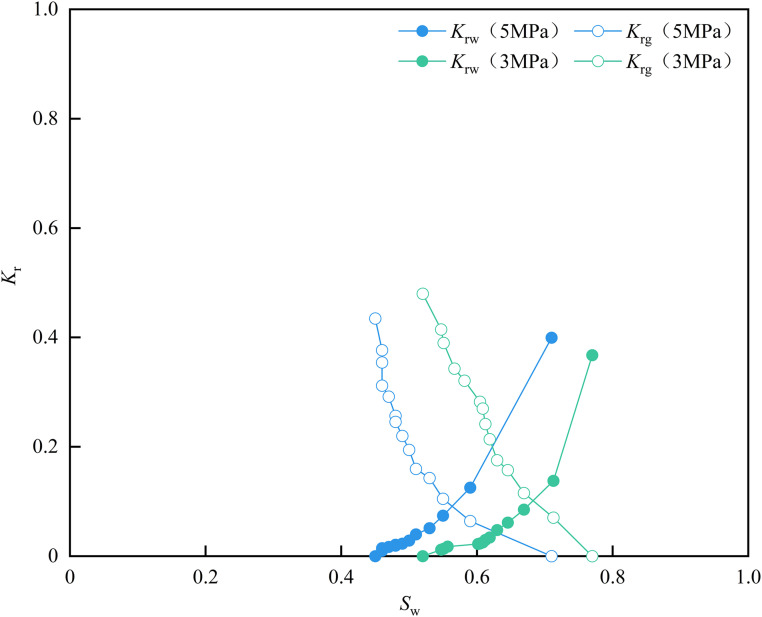
Gas-water relative permeability curves of matrix-fracture type samples.

As shown in Fig 15, as the displacement pressure increases, the water phase flow capacity increases. Under the same water saturation condition, the higher the water phase relative permeability, the lower the irreducible water saturation from 0.52 to 0.452, while the co-permeability zone increases slightly from 0.25 to 0.255. This is because the reduction in effective stress, which promotes fracture expansion and increased aperture, thus enhancing the flow capacity of the sample [25]. Meanwhile, the higher displacement pressure can overcome the greater capillary force, effectively displacing the irreducible water in the smaller pores. With the increase of displacement pressure, the equal permeability point shifts to the lower left, and the corresponding water saturation is greater than 0.5, indicating that the experimental samples are hydrophilic. In addition, with the increase of displacement pressure, the gas phase relative permeability is lower under the same water saturation condition. This phenomenon occurs because the increased displacement pressure reduces the thickness of the irreducible water film, resulting in an increase in the movable water volume and an increase in the gas phase flow resistance under the same water saturation. The parameters of the gas-water relative permeability curve are detailed in Table 3.

**Table 3:** Gas-water relative permeability curves parameters of matrix-fracture type samples.

3MPa			5MPa		
*S*_w_	*K*_rw_	*K*_rg_	*S*_w_	*K*_rw_	*K*_rg_
0.520	0.000	0.480	0.452	0.000	0.435
0.547	0.012	0.414	0.456	0.010	0.377
0.551	0.014	0.390	0.459	0.011	0.354
0.567	0.018	0.343	0.464	0.015	0.312
0.582	0.022	0.321	0.470	0.017	0.292
0.605	0.023	0.282	0.476	0.019	0.257
0.609	0.025	0.270	0.483	0.021	0.245
0.613	0.030	0.242	0.490	0.023	0.220
0.619	0.034	0.214	0.499	0.028	0.194
0.630	0.048	0.175	0.512	0.040	0.159
0.645	0.062	0.157	0.529	0.051	0.143
0.669	0.085	0.116	0.553	0.074	0.105
0.713	0.138	0.071	0.594	0.125	0.064
0.770	0.367	0.000	0.707	0.400	0.000

**Fig 16:**
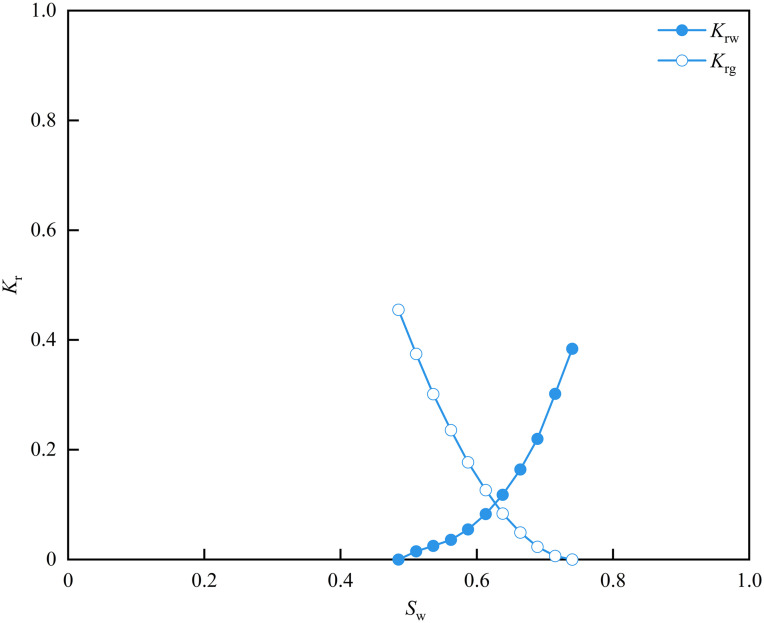
Normalized gas-water relative permeability curves of matrix-fracture type samples.

In numerical simulation studies of oil and gas reservoirs, the measured curves need to be normalized. Therefore, the direct average method is applied to normalize the gas-water relative permeability curve of the shale in the research area. The detailed processing steps have been presented in previously published articles and will not be reiterated here [26]. As shown in Fig 16, the normalized gas-water relative permeability curve closely matches the measured curve, relatively reflecting the gas-water flow characteristics. The specific parameters are detailed in Table 4.

**Table 4:** Normalized gas-water relative permeability curves parameters of matrix-fracture type samples.

*S*_w_	*K*_rw_	*K*_rg_
0.486	0.000	0.458
0.511	0.015	0.375
0.536	0.025	0.301
0.562	0.036	0.236
0.587	0.055	0.177
0.613	0.083	0.127
0.638	0.118	0.084
0.664	0.164	0.049
0.689	0.220	0.023
0.715	0.302	0.006
0.739	0.384	0.000

A reverse gas displacement experiment was conducted on matrix-type shale sample 6# under initial displacement pressure of 30 MPa and confining pressure of 35 MPa. However, after 240 hours, no gas was observed. Subsequently, the displacement pressure was gradually increased in 5 MPa intervals, with the confining pressure maintained 5 MPa higher than the displacement pressure throughout. Under displacement pressures of 35 MPa, 40 MPa, 45 MPa, and 50 MPa, the gas production remained undetected after 120 h of reverse gas displacement at each pressure. Due to the maximum pressure limit of 50 MPa for the intermediate container in the laboratory setup, further increase in displacement pressure was not feasible. Therefore, it is concluded that gas cannot break through the matrix-type sample. The reason is the extremely low permeability of the matrix-type sample, and effective gas-water two-phase flow cannot be achieved without fractures. Similarly, sample 4# also failed to exhibit gas breakthrough under an initial displacement pressure of 25 MPa, further supporting this inference.

## 4. Conclusions

To reveal the gas-water two-phase seepage law in shale matrix-fracture systems and clarify its gas-water two-phase flow characteristics, water displacement gas and reverse gas displacement water experiments were conducted on matrix-type and matrix-fracture type samples under different displacement pressures. The main understanding is as follows:

(1) The matrix-type samples did not exhibit formation water breakthrough, with the displacement velocity gradually decreasing over time. During the 408 ~ 504 h interval, the displacement velocity approached zero, and the NMR signal stabilized. The higher the experimental displacement pressure, the faster the displacement velocity, the deeper the formation water invades the matrix-type samples, the higher the NMR signal intensity, and the higher the water saturation.
(2) The matrix-fractured sample breaks through in a short time, and the NMR signal is stable during the 24 ~ 28 h interval, requiring significantly less time than the matrix-type samples. The higher the experimental displacement pressure, the faster the displacement velocity, the lower the NMR signal and the lower the water saturation. Under the same displacement pressure, the NMR signal intensity of matrix-fractured samples is higher than that of matrix-type samples.
(3) The *T*₂ spectrum of the matrix-type samples exhibited a unimodal distribution. As displacement time increased, formation water first filled the micropores, then the mesopores, and occurred on the pore surfaces in the form of a “thick water film” or a “water column.” Higher displacement pressures resulted in higher *T*₂ peak values, greater signal intensity in the same section, and deeper invasion depth. The *T*₂ spectrum of the matrix–fracture type samples showed a bimodal distribution (with the left peak higher than the right), and the filling law and occurrence form of formation water were consistent with those observed in matrix-type samples. With increasing displacement pressure, the left *T*₂ peak value decreased, and the signal intensity in the same section was also lower.
(4) With increasing displacement pressure, the water phase flow capacity increases. Under the same water saturation condition, the higher the water phase relative permeability, the lower the irreducible water saturation, decreasing from 0.52 to 0.452, while the co-permeability zone increases slightly from 0.25 to 0.255. The equal permeability point shifts to the lower left, and the corresponding water saturation is greater than 0.5, indicating that the experimental samples are hydrophilic. In addition, under the same water saturation condition, the gas phase relative permeability is lower. However, the reverse gas displacement failed to achieve a breakthrough in the matrix-type samples from the research area, indicating that the permeability of the matrix-type samples is too low, resulting in the gas-water two-phase seepage state not being achieved without the presence of fractures.

## Supporting information

S1 File Supporting information. (ZIP)
